# Interrelationship of Pyrogenic Polycyclic Aromatic Hydrocarbon (PAH) Contamination in Different Environmental Media

**DOI:** 10.3390/s91209582

**Published:** 2009-11-30

**Authors:** Seung-Kyu Kim, Dong Soo Lee, Won Joon Shim, Un Hyuk Yim, Yong-Seung Shin

**Affiliations:** 1 School of Earth and Environmental Sciences (BK-21), Seoul National University, Kwanakgu Kwanakro 599, Seoul 151-742, South Korea; E-Mail: skkim89@gmail.com; 2 Graduate School of Environmental Studies, Environmental Planning Institute, Seoul National University, Kwanakgu Kwanakro 599, Seoul 151-742, South Korea; 3 Oil & POPs Research Group, Korea Ocean Research and Development Institute, 391 Jangmok-ri, Jangmok-myon, Geoje-shi 656-834, South Korea; E-Mails: wjshim@kordi.re.kr (W.J.S.); uhyim@kordi.re.kr (U.H.Y.); 4 Environmental Policy Division, Korea Environment Institute, Eunpyung ku, Bulkwang dong 613-2, Seoul 122-706, South Korea; E-Mail: shiny@kei.re.kr

**Keywords:** PAHs, fugacity ratio, multi-media fate, partitioning equilibrium, black carbon

## Abstract

Interrelationships between pyrogenic polycyclic aromatic hydrocarbons (PAHs) were assessed in air, soil, water, sediment, and tree leaves by using multi-media monitoring data. Concurrent concentration measurements were taken bimonthly for a year for the multi-media at urban and suburban sites. PAH level correlations between air and other media were observed at the urban site but were less clear at the suburban site. Considering a closer PAHs distribution/fate characteristics to soil than suspended solids, contamination in sediment seemed to be governed primarily by that in soil. The partitioning of PAHs in waters could be better accounted for by sorption onto black carbon and dissolved organic carbon.

## 1. Introduction

In urban and suburban areas, polycyclic aromatic hydrocarbons (PAHs) are often of pyrolytic origin and initially enter into air. They are subsequently re-distributed in the multiple environmental media (e.g., air, water, soil, and sediment) as governed by their fate properties and environmental and meteorological conditions. Therefore, the total risk posed by PAHs in an environmental system comprising multiple media could properly be assessed when the extents of PAH contamination in all the multi-media are simultaneously taken into account. However, such total risk assessment has been limited because either the concentration was often measured for a single medium or two [1-4] or when the multi-media data were available, the measurements were not taken in a concurrent manner [5]. Due to the lack of the measured data, multi-media models often were used as an alternative for the assessment [6]. Use of the models, however, has also been limited largely because of prediction uncertainty [7-9]. The prediction uncertainty could eventually be assessed and/or reduced by comparing the model predictions with the measured data [10-12] if the data are available. Therefore, regardless of whether the measured data or multi-media models are used for total risk assessment, it is of critical significance to fill the data gap for the measured multi-media concentrations.

The objectives of the present work were to provide a concurrently measured multi-media data set and to obtain a total picture of PAH contamination in a multi-media environmental system by assessing the data. Of particular concern was the assessment of the interrelationship of the PAH levels among the various media (*i.e.*, influence of the contamination level particularly in air on those in other media and extent of deviation from the chemical equilibria among the media) under the field conditions. To our knowledge, this kind of extensive concurrent measurement work including air, water, soil, plant leaf, atmospheric dry deposition flux, and size-segregated PM is scarce and therefore considered valuable.

## 2. Experimental Section

### 2.1. Study Site

Seoul is a metropolitan city with the area of 605.5 km^2^. Annual average wind speed is 2.0 ± 0.4 m/s. Annual average precipitation and the number of rainy day are about 1,350 mm and 100 days, respectively. Over 70% of the precipitation and 40% of the rainy day occur from June to August. The rainfall intensity over 10 mm/day, which yields runoff, occurs mostly in the wet period. The Han River flows through the mid-part of Seoul to the Yellow Sea at average flow rates of 2 m/s in summer and 0.1 m/s in winter.

**Figure 1.**
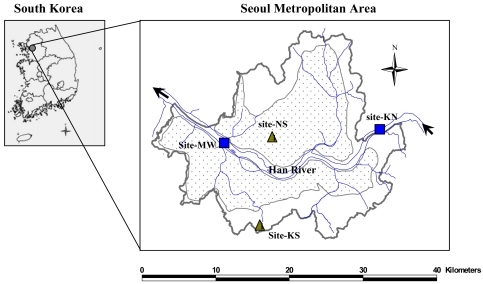
The study area and the sampling locations (NS and KS for terrestrial samples, KN and MW for aquatic samples). Dotted area represents the population-dense area.

Sampling was conducted in the urban (NS and MW) and the suburban (KS and KN) areas (Figure 1). The sites of NS and KS (12.5 km apart) were used for terrestrial sampling while KN and MW (22.3 km apart) were for the aquatic sampling. The depths at KN and MW were about 2 m and 10 m, respectively. Six tributaries merge into the mainstream between KN and MW.

### 2.2. Sample Collection

Concurrent sampling was conducted bimonthly for multiple media from August 2001 to April 2002. The environmental and weather conditions are summarized in Table 1. The sampled media were air (vapor and particulate phases), water (dissolved and suspended solid phases), sediment, soil, and coniferous and deciduous leaves, and dry deposition flux of PM. Air was sampled at a rate of 600 L/min using high volume (Hi-vol) air samplers (Kimoto Model-123V and Kimoto Model-120FT at KS and NS, respectively) with glass fiber filters (20.3 × 25.4 cm, EPM-2000, Whatman) and two serially loaded polyurethane foam (PUF) plugs (6 cm O.D. × 7.6 cm length, Supelco). For size-segregated sampling of PM, a cascade impactor (CI, Andersen, SA236) was used together with Hi-vol sampler at both KS and NS. Dry deposition flux was also measured using three dry deposition plates with four Mylar strips (Graphic Art Systems, USA, 5.7 cm × 1.8 cm) coated with Apezion L grease (thickness; ∼5 μm) [13]. To further evaluate the effects of different air sampling techniques (*i.e.*, Hi-vol versus CI (or MOUDI)), additional samples using the Micro-Orifice Uniform Deposit Impactor (MOUDI, MSP Corporation, USA) and Hi-vol sampler were collected at both the sites every month from December 2002 to March 2003.

The surface soil (5 cm) was taken using a stainless steel shovel pre-cleaned with dichloromethane (DCM) and sieved through 2 mm opening into pre-cleaned amber bottles. Pine (*Pinus rigida*) needles and Mongolian oak (*Quercus mongolica*) leaves were cut-collected using DCM pre-cleaned stainless steel tweezers. The leaf surface was washed off using hexane cleaned water before analysis.

Surface water of 20 L was taken into DCM pre-cleaned 4 L amber bottles at a depth of 10 cm. Suspended solids (SS) were seperated using 0.7 μm glass fiber filter (GFF, Whatman) and kept at –20 °C until analysis. The dissolved phase was acidified with hydrochloric acid to maintain the pH below 2 and stored at 4 °C. Surface sediment (about 2 cm) was taken using a stainless grab sampler at the water sampling location and kept at –20 °C until analysis. Except air samples, a composite sampling technique (*i.e.*, combining five space-scaled samples at the same mass quota) was applied to improve the spatial representation of the measurements.

### 2.3. Chemical Analysis and QA/QC

A total of 24 PAH compounds were analyzed following a modified NOAA method [14] for solid samples and [15] for water samples. All extracts in *n*-hexane were cleaned by silica/alumina packed column and, if necessary, further using Phenogel 100 Å size-exclusion high performance liquid chromatography (Phenomenex, Rancho Palos Verdes, CA, USA). PAH compounds were analyzed using a 5890GC/HP5973A GC (Hewlett-Packard, USA) equipped with a 30 m × 0.25 mm I.D. DB-5 capillary column (J&W Scientific, Folsom, CA, USA) under scheduled temperature operating conditions described elsewhere [7].

For analytical quality control purposes, a procedural blank, a standard reference material (or fortified sample), and a duplicate sample were run together in each analysis batch (12–24 samples) and a field blank sample collected every sampling event was also analyzed. Target compounds in the two-type blank samples were always below three times method detection limits (MDL). All the reported values in the present study are blank-subtracted ones. Standard reference materials (SRMs) of NIST 1941a (National Institute of Standards and Technology, Gaithersburg, MD, USA) and IAEA-142/OC (International Atomic Energy Agency, Vienna, Austria) were analyzed to confirm the accuracy of analytical method for solid samples. Fortified water samples spiked in known PAHs concentration were analyzed for water samples. Analytical mean values of all PAHs in these SRMs and fortified samples fell within 40–120%. However, nine low molecular weight PAHs (LPAHs) including naphthalene, 2-methylnaphthalene, 1-methylnaphthalene, biphenyl, 2,6-methylnaphthalene, acenaphthylene, acenaphthene, 2,3,5-trimethylnaphthalene, and fluorine were not quantified and included for data analysis of both dissolved and particulate phases due to their low recovery and of gaseous phase due to break-though. Except for these three phases, recoveries of surrogate standards were within the acceptable range (e.g., average 68.6% for naphthalene-*d8*, 74.0% for acenaphthene-*d10*, 85.4% for phenanthrene-*d10*, 92.7% for chrysene-*d12*, and 72.5% for perylene-*d12*) for the others.

### 2.4. Organic Carbon Analysis

For organic carbon content analysis, all solid samples were freeze-dried and then were ground using a mortar. Inorganic carbon in the samples was removed by treating with 10% (v/v) hydrochloric acid and dried again in a 50 °C oven overnight. The powdered samples were analyzed using a TOC analyzer (SSM-5000A, Shimadzu, Japan). Organic carbon contents in the dissolved phase in water were also measured using the same instrument equipped with a liquid module.

## 3. Results and Discussion

### 3.1. TPAHs Concentration in Multi-Media

The concentrations of total PAHs (TPAHs) and of individual PAHs in each medium are presented in Tables 1 and 2, respectively. The TPAHs levels were generally greater at the urban sites than the suburban sites. Compared with the cities of other countries, the mean atmospheric TPAHs in Seoul was similar to those in London, Jakarta, and Chicago and was lower than those in the cities of eastern China [16-18]. The levels of TPAHs in other media of Seoul appeared also lower than those in the cities of eastern China [19-21].

### 3.2. Atmospheric Fate of PAHs

The study of particular signature PAHs [22] indicated that PAHs in this area were principally pyrogenic (*i.e.*, PH/AN < 10 and FRL/PY > 1). As pyrogenic PAHs would enter into the atmosphere first and subsequently transfer to other environmental media, the levels and fates in the atmosphere should strongly influence those in other media.

**Figure 2.**
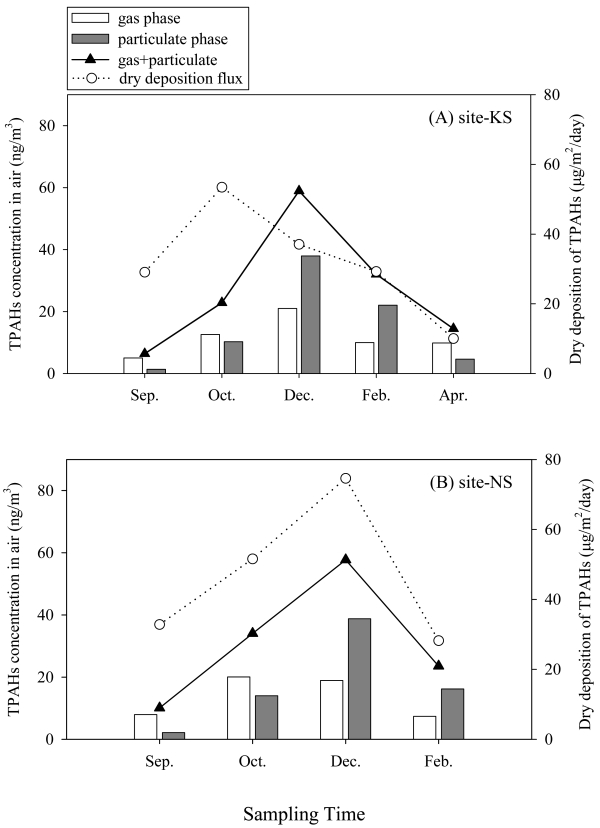
Temporal variation of atmospheric TPAHs concentrations and dry deposition flux of particulate TPAHs measured at (A) suburban and (B) urban sites. TPAHs denotes the sum of gaseous and particulate PAHs for 15 compounds (listed in Table 2).

The atmospheric levels of TPAHs (the sum of 15 PAHs in both gas and PM) in air were similar at the two sites, ranging from 10 ng/m^3^ to 58 ng/m^3^ at the urban site (NS) and from 6.3 ng/m^3^ to 59 ng/m^3^ at the suburban site (KS). The TPAHs increased in winter at both sites (Figure 2), which was attributable to increased fuel combustion (70% of the total PAH emission) for heating [16] and the reduced wind speed [23,24]. Gaseous PAHs were dominant in the warm periods while particulate PAHs were so in the cold season likely due to temperature effect.

**Figure 3.**
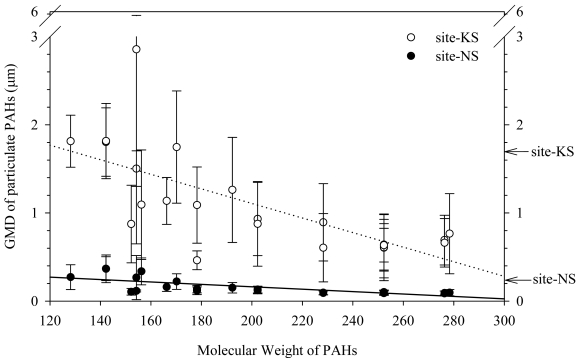
Variation of geometric mean diameter (GMD) of particulate PAHs in air. (log*GMD* = Σ*m_i_*log*Dp_i_*/Σ*m_i_*, where *m_i_* and *Dp_i_* are the mass of individual PAHs and the geometric mean particle diameter, respectively, of size class i). Circles and error bars denote annual mean and one standard deviation of GMDs, respectively. Arrows at right axis represent the annual GMDs of PM at KS (1.7 ±1.0 μm) and at NS (0.23 ± 0.15 μm).

The mass fraction of fine PMs (<3 μm) was predominant at the NS site (average fraction ± one standard deviation (STD) = 88% ± 8%) while that of coarse PMs (>3 μm) was significant at the KS site (average fraction ± STD = 36% ± 19%). Consequently, the PM size distributions of PAHs were significantly different at the two sites (paired t-test, p < 0.05) (Figure 3). More PAHs were associated with fine PMs at the NS site than at the KS site. Consistently, the mass fraction of high molecular weight PAHs (HPAHs, molecular weight over 202) was greater at the NS site than at the KS site (annual averages of 73% at NS and 54% at KS) because of HPAHs' greater affinity to finer PMs [26]. It was further noted that the seasonal variations of geometric mean diameter (GMD) of PM and the PM bound individual PAHs were greater at KS than at NS as depicted by the error bars in Figure 3.

**Figure 4.**
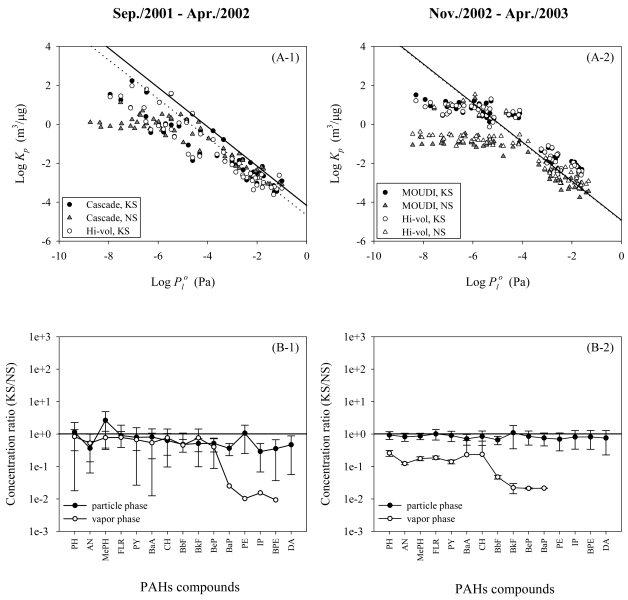
Comparison of (A) partition coefficients (K_p_) of PAHs observed at suburban (KS, circles) and urban (NS, triangles) sites (Dotted and solid lines denote the mean K_p_ values predicted from Junge-Pankow model (Atmos. Envion. 1987, 21, 2275-2283) for KS and NS, respectively.) and (B) concentration ratio between the two sites.

The observed partition coefficient, K_p_ = (C_p_/TSP)/C_g_, where C_p_ and C_g_ are PAHs concentrations in PM and gas phases, respectively, and TSP denotes concentration of PM), was greater for finer PMs and at lower temperature at each of the sites. However, the gas-PM partitioning appeared unusual in that the K_p_ values for HPAHs were smaller at the NS site (where fine PMs were dominant) than at the KS site. Furthermore, as shown in Figure 4(A-1), the log K_p_ values of the HPAHs appeared to level off with decreasing vapor pressure. Similar data have been presented in some studies [27,28] although little attention was paid to the presence of the leveling off phenomenon. Such deviation from gas-PM partitioning theories has often been ascribed to sampling artifacts [29,30].

To examine the possibility of the sampling bias, a total of four additional sample sets of PM and gas were collected from December, 2002 to March, 2003 by using the same techniques at the two sites deploying both the MOUDI and Hivol-PUF samplers. As shown in Figure 4(A-1) and 4(A-2), the difference in the sampling technique (*i.e.*, CI versus Hi-vol or MOUDI versus Hi-vol) appeared to have little influence on the results. The site difference in the K_p_ values and the leveling-off phenomenon for the HPAHs were even more clearly observed.

The smaller values of K_p_ for HPAHs at the NS site resulted because the PM bound concentrations (in ng/g) were similar at the two sites while the gas phase concentrations were greater by two orders of magnitude at NS for HPAHs [Figures 4(B-1) and 4(B-2)]. In the source area, the emission of gaseous HPAHs was likely to be stronger than in the suburban area. Theoretically, if the contact time for the gaseous PAHs to sorb onto PMs was insufficient in the source area due to their slow sorption kinetics [31] and/or if no more sorbent surface was available due to saturation, the smaller value of K_p_ could occur.

### 3.3. Influence of Atmospheric TPAHs Level on the Other Media

**Figure 5.**
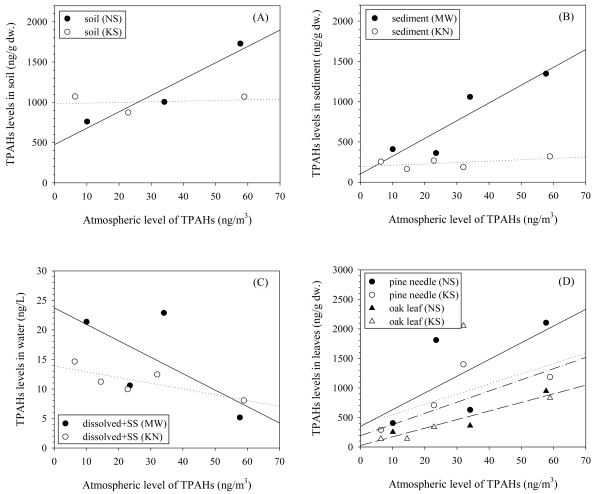
Correlation of atmospheric level of TPAHs (gas + particulates) with that in (A) soil, (B) water (dissolved + suspended solids), (C) sediment, and (D) leaves. TPAHs is the sum of 15 PAHs (PH∼DA of MW > 178 in Table 2) for air and water and the sum of all 24 PAHs for the others.

The relationship of TPAHs in air and those in soil and sediment varied with the site. A clear positive and linear correlation was observed at the source site (NS), while no significant correlation appeared at the suburban site (KS) [Figures 5 (A) and (B)]. TPAHs in water and plant leaf showed a negative and positive relationship with those in air, respectively, at both sites [Figures 5 (C) and (D)].

Both field measurements and model estimation by POPsME [7] indicated that the deposition of PM bound PAHs was the most significant cross-media process at the two sites. The deposition is a strong function of both the PM size distribution [32] and the atmospheric PAHs level. As noted earlier in Figure 3, the seasonal variations of geometric mean diameter (GMD) of individual PAHs and PMs were greater at KS than at NS. This condition at the KS site suggests that the input of PAHs to soil would significantly be influenced both by the atmospheric level and the variation of the PM size distribution at the site. Contrarily, the input at the NS site might principally be governed only by the atmospheric PAHs level because the PM size distribution was fairly constant over the year. Evidently, the measured dry deposition flux at NS was in better correlation with the atmospheric PAHs level (Figure 2) as compared to the KS site. However, the contamination level in soil or sediment has been known to slowly respond to the change in the atmospheric contamination level [4]. Although observed at the urban site, the correlation between the PAHs level in air and that in soil might have occurred by chance. Further investigation is necessary to corroborate the explanation for the observed correlation. It is shown in Figure 5(B) that the level of TPAHs in sediment was well correlated with that in air at the urban area, suggesting that the main inputs of PAHs to sediment might occur directly from air and/or via the route of air to soil to sediment. Based on observed PAHs composition profile, fugacity, and organic carbon fraction, further discussion is given in the next sections concerning the principal pathways of chemical input to sediment.

The absence of a positive correlation was anticipated between air and water because the PAH concentration at a certain point or section in the river water is governed not only by the input from air but those from upstream, merging tributary streams, surface run-off, and sediment. Besides, the concentration in the river water strongly depends on the volumetric flow rate of the stream. The contamination levels in the plant leaves clearly reflected the variation in atmospheric concentration, which is consistent with the known uptake mechanisms [33].

### 3.4. PAH Profiles in Solid Media

**Figure 6.**
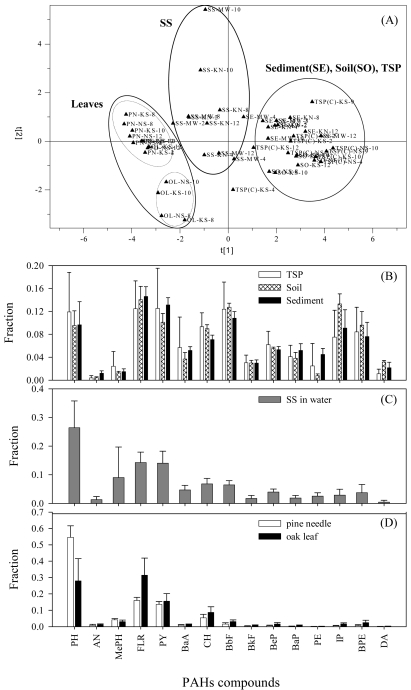
PAH profiles in the environmental solid media. (A) grouping of the PAHs profiles by principle component analysis (PCA), (B) profiles in PM, soil, and sediment, (C) profile in SS (NA: not analyzed), and (D) profiles in the leaves. PCA was conducted for 15 PAHs (listed in Table 2) where primary and secondary components explained 66% of total variation.

The mass fraction of individual PAHs to TPAHs in environmental solids generally increases with the hydrophobicity [34]. However, specific PAH profiles varied with the kind of the environmental solids (Figure 6). By principal component analysis (SIMCA 6.01, Umetri AB, USA) of the PAH profiles, the solid media could be divided into three groups. Soil, sediment and PM showed relatively similar profiles where HPAHs were abundant. However, the fractions of HPAHs were substantially lower in SS as compared to the group comprising soil, sediment and PM. The plant leaves were characterized with the profiles of no or extremely low fractions of HPAHs.

**Figure 7.**
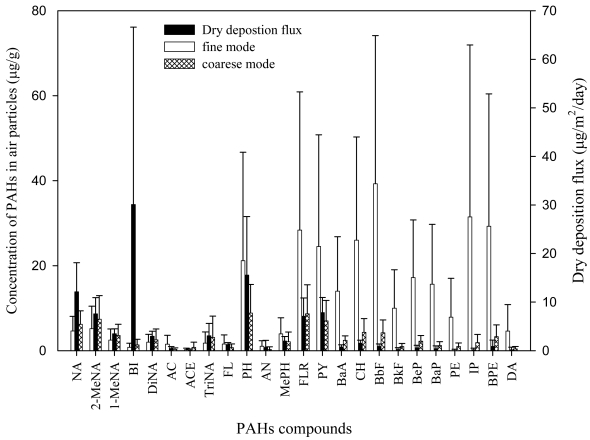
Annual average concentration of PAHs in PM (fine mode: <3 μm, coarse mode: >3 μm) and dry deposition fluxes. Error bars denote one standard deviation.

The discrepancy in the PAH profiles between SS and their source and sink media (soil, PM, and sediment) presented valuable information on the possible pathways of PAHs into water and partitioning onto SS. Some researchers have also reported the comparatively low fractions of HPAHs in SS [35,36]. The origin of SS includes PM, eroded soil particles, re-suspended sediment particles, and the biological particles. As the PAH profiles in PM, soil, and sediment appeared relatively similar to each other, the biological particles were likely responsible for the observed difference in the PAHs profile in SS. The relative mass contributions of the biological particles to SS vary with season. In the present study, a significant increase in the organic carbon fraction (F_oc_) of SS was observed during an algal blooming period (F_oc_ = 0.21, often warm late winter to early spring in this area) as compared to rainy summer season (F_oc_ = 0.05). During the rest of the dry season, the principal contributors to SS appeared to be PM (F_oc_ of 0.12 to 0.3) and/or phytoplankton (F_oc_ = 0.27 ± 0.14, not published) because annual average F_oc_ of SS (0.126 ± 0.067) was significantly greater (p < 0.05) than those of soil (0.047 ± 0.010) and sediment (0.016 ± 0.004). Thus, the small HPAHs-fraction observed in SS might be a result of introduction of the biological particles in significant portion because slow sorption-desorption kinetics as compared to the algal growth rate could lead to the apparent lack of the more hydrophobic compounds in SS [37]. Size dependent deposition velocity of PM could in part account for the lower HPAHs in SS. The PAH profiles of dry deposition flux resembled those in coarse PMs (Figure 7), suggesting that PAHs on coarse PMs deposited faster. The fractions of HPAHs tended to be smaller in coarse PMs than in fine ones in the present study as well as others [25,38]. Consequently, the relative depletion of HPAHs (as compared to PM) could be reflected both in soil and SS to which deposition of PM-bound PAHs is a major input pathway. In soil surface, however, comparatively active evaporation and degradation of the lighter PAHs [39] is likely to lead to the abundant fractions of HPAHs. Under the condition that the transfer of HPAHs is directed from atmospheric gas to the dissolved phase and then further to SS, slow diffusion kinetics of the HPAHs could result in their relative depletion in SS. Dissolved organic carbon might also contribute to the depletion of HPAHs in SS by competitive sorption of the HPAHs as its level was significant (10.9 ± 8.5 mg/L) at the sites.

In sediment, the PAH profile was similar to that in soil. Further, the temporal and spatial variations of TPAHs level, PAHs profile, and F_oc_ in sediment were not comparable with those in SS at the same locations in the river. Such observations suggested that a primary origin of sediment might be heavier and fast settling soil particles that were delivered to the river. Therefore, it should be noted that a modeling concept adopting a simple vertical exchange of particles between sediment and SS in overlying water column could result in large prediction bias for the river system.

### 3.5. Phase Equilibria in Multimedia Environment

Fugacity [42] was compared to assess whether the phase equilibrium has been established between different pairs of environmental phases. Fugacity in a medium *i* was calculated by the equation: *f_i_* = *C_i_/Z_i_*, where f_i_, C_i_, and Z_i_ is fugacity, concentration, and fugacity capacity, respectively. To calculate Z values, temperature dependent vapor pressure and Henry's law constant were estimated following [43] and [44], respectively. Among the various forms of carbons as effective sorbents, only organic carbon (OC) was taken into account in the calculation of fugacities [42].

**Figure 8.**
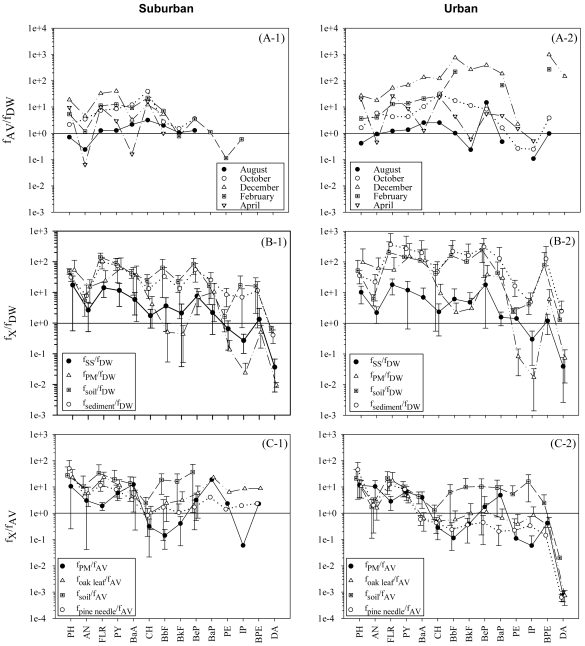
Comparison of the fugacity (*f*) among environmental media. Error bars denote one standard deviation of seasonal geometric means. The subscripts, AV, DW, X, SS and PM, denote vapor phase in air, dissolved phase in water, environmental solids, suspended solids in water, and PM, respectively.

It is shown in Figures 8(A-1) and 8(A-2) that greater f_AV_ (AV: vapor in air) than f_DW_ (DW: dissolved phase in water) except in August, indicating that chemical equilibrium might establish only for a limited duration of the year. The deviation from equilibrium was greater at the urban site than the suburban site probably because the emission rate of PAHs into air was larger at the urban site, allowing less time to equilibrate with other media. Consistently, the deviation of f_AV_/f_DW_ from unity (*i.e.*, equilibrium) became greater in December probably due to increased emission of PAHs into air in winter.

As shown in Figures 8(B-1) and 8(B-2), fugacity of PAHs was greater in various environmental solid phases than that in the dissolved phase except for a few HPAHs. As f_SS_ was greater than f_DW_, the net flux of PAHs was considered to occur from SS to the dissolved phase for most PAHs. However, it should be noted that f_SS_ was calculated under an assumption that organic carbon is the dominant sorbent. If other strong sorbents (e.g., black carbon (BC)) were available [44], f_SS_ would substantially be smaller and the direction of PAH transfer could vary for most PAHs (see the section 3.6 for further discussion).

Figures 8(C-1) and 8(C-2) compare the fugacity in the environmental solid phases to f_AV_. Fugacity for most PAHs was similar among the vapor phase, PM, and leaves, indicating that these were in or near equilibrium. Fugacity in soil, however, appeared far greater than that f_AV_ and f_PM_, clearly showing that the input of PAHs from air to soil was driven not by the chemical gradient but principally by the physical processes such as dry and wet PM depositions. Compared with concentrations observed 10 years ago in Seoul [16], individual PAHs in PM decreased significantly. Thus, the greater fsoil than f_AV_ or f_PM_ might result from historical accumulation in soil due to higher atmospheric levels in the past [4]. In summary, chemical equilibrium was likely only among vapor, PM, and leaves for some PAHs and, for a limited period, between vapor and the dissolved phase.

### 3.6. Black Carbon Effect on Partitioning and Fate

**Figure 9.**
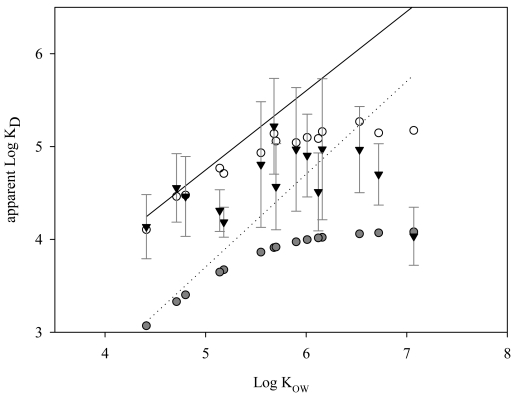
Apparent distribution coefficients (K_D_) between SS and water with respect to K_OW_: observed K_D_ versus estimated K_D_ from Equation 1 and 2 in the text. Error bars, gray-filled ((x025CF)) and open circles, and triangles ((x025BC)) denote one standard deviation, average K_D_s estimated by Equation 1 and 2, and observed values in this study, respectively. Dotted and solid lines represent K_D_s estimated by Equation 1 and 2 with DOC value set to zero, respectively.

In the present study, fugacities of SS and sediment appeared to be greater than that in the dissolved phase, which corresponded to more than one order of magnitude greater solid-water partition coefficients (K_D_) than those expected from the OC partitioning model. Such elevated partition coefficients were frequently observed between aquatic solids and water [45-48]. To account for the elevated values of K_D_, effects of DOC and/or BC were included in the partitioning models as expressed by Equation1 [49] or 2 [50] below:

(1) $$K_{D}^{\text{app}}=\frac{F_{\text{OC}}\times K_{\text{OC}}}{1+K_{\text{DOC}}\times (\text{DOC}\times 10^{-6})}$$

(2) $$K_{D}^{\text{app}}=\frac{F_{\text{OC}}\times K_{\text{OC}}+F_{\text{BC}}\times K_{\text{BC}}\times C_{\text{DW}}^{n-1}}{1+K_{\text{DOC}}\times (\text{DOC}\times 10^{-6})}$$

where F_OC_ and F_BC_ are the fractions of OC and BC in SS, respectively, and K_OC_ (L/Kg), K_BC_ ((L)^n^/Kg), and K_DOC_ (L/Kg) denote the partition coefficients between the freely dissolved phase and OC in SS, BC in SS, and DOC, respectively. C_DW_ (μg/L) and DOC (mg/L) are the concentrations of compound in freely dissolved water and concentration of DOC in water column, respectively, and n is Freundlich exponent. When applying the observed averages of F_OC_ and DOC in the present study, K_DOC_ ≈ 1 × K_OC_ (=0.41K_OW_) [51], F_BC_ reported in the field (*i.e.*, 0.003) [8,45,46], n (*i.e.*, 0.7) and K_BC_ in literature [52], the K_D_ values determined by Equation 2 were comparable to the observed average values within a factor of three while those by Equation 1 were different from the observed values by 1 to 2 orders of magnitude (Figure 9). Therefore, in addition to OC in SS, both BC (for most PAHs) and DOC (for HPAHs of log K_OW_ > 5.5) might substantially have contributed to the partitioning of PAHs in water column.

Recent BC-inclusive multimedia fate models [47,48] also predicted 1-2 orders of magnitude lower dissolved concentration than estimates of BC-exclusive model. According to those models, BC-inclusion effect in terms of multimedia fate was evident, particularly for LPAHs, because the advective losses out of the model domain were reduced as a result of the increase of sorption to SC of sediment and SS although air levels or deposited amounts to soil were little affected. Therefore, it might be essential to add the sorption to BC in future multimedia fate and exposure models.

## 4. Conclusions

The level of contamination showed no positive correlation between air and water, while tree leaves were found to have good correlation with the atmospheric PAH level. The influence on soil of the atmospheric level of PAHs was observed to appear more clearly when the PM size distribution was relatively constant in time. The environmental solids such as PM, soil and sediment were closely related to each other for their contamination level and PAHs profile. Particularly, the observed levels and profiles of PAHs strongly suggested that the contamination of sediment originated from that of soil. The PAHs level and profile in SS, however, appeared to be interfered with by the biological particles produced within the water column and/or DOC sorption, showing little similarities of contamination with other environmental solids. Within the multimedia environment system in the present study, fugacity comparison indicated that non-equilibrium conditions prevailed except for certain LPAHs in vapor, PM, and plant leaves for limited time period. Calculations suggested that the potential role of BC and DOC should further be investigated to better judge the partitioning equilibrium relationship between the dissolved phase and SS in water. Furthermore, BC and DOC could become critical parameters determining the level in the dissolved phase and SS and thus residence time and bioavailability. Implications to modeling were that frequent use of the OC dominating sorption model in existing multimedia models could cause significant prediction biases for the concentrations in the media involving the carbons as sorbents. The prediction bias might further magnify for f_sediment_/_fpore water_ or f_sediment_/f_SS_ if sediment is modeled to form primarily by vertical settling of SS from the overlying water column. As observed in the present study, fugacity and the composition profile was significantly different between sediment and SS. Quantitative investigation of biological particles and black carbon in water and in-depth understanding of SS settling and sediment transport appear critical to improving the model's capability to predict the fate and transport of PAHs in aquatic systems.

## Figures and Tables

**Table 1.** Environmental and weather conditions during sampling and TPAHs^a^ concentration in concurrently measured multi-media environmental samples.

	**Unit**	**Sampling sites**	**Aug. (or Sep.***^c^***) 2001**	**Oct. 2001**	**Dec. 2001**	**Feb. 2002**	**Apr. 2002**
**Environmental parameters**							
**Air temp.**	°C	average	23	17	-4.3	3.0	7.7
**Water temp.**	°C	average	26	18	0.60	5.7	9.7
**SS conc.**	(mg/L)	MW	23	20	9.0	24	34
		KN	21	9.8	3.9	7.2	18
**TSP conc.**	(μg/m^3^)	NS	32	50	60	55	83
		KS	44	220	98	150	130
**SS_Foc**	dimensionless	MW	0.067	0.069	0.094	0.18	0.18
		KN	0.052	0.12	0.18	0.25	0.070
**Sediment_Foc**	dimensionless	MW	0.013	0.018	0.017	0.014	0.025
		KN	0.011	0.014	0.015	0.020	0.015
**Soil_Foc**	dimensionless	NS	0.046	0.035	0.057	0.064	0.055
		KS	0.035	0.041	0.040	0.052	0.049
**TPAHs concentration in each medium**							
**Air***^b^*	(ng/m^3^)	NS	10	34	58	24	-- *^d^*
		KS	6.4	23	59	32	15
**Dry deposition flux**	(μg/m^2^/day)	NS	80	140	130	66	-- *^d^*
		KS	75	93	68	56	79
**Soil**	(ng/g dw.)	NS	760	1000	1700	--*^d^*	-- *^d^*
		KS	1100	870	1100	-- *^d^*	-- *^d^*
**Leaves**	Pine needle (ng/g dw.)	NS	400	630	2100	1800	-- *^d^*
		KS	290	710	1200	1400	-- *^d^*
	Oak leaf (ng/g dw.)	NS	250	360	950	-- *^d^*	370
		KS	140	340	830	2100	140
**Water***^b^*	(ng/L)	MW	21	23	5.2	11	32
		KN	15	9.9	8.0	13	11
**Sediment**	(ng/g dw.)	MW	410	1100	1300	360	510
		KN	250	270	320	190	160

a Sum of concentrations of 24 PAHs listed in Table 2.

b Sum of vapor and particulate PAHs in air and sum of dissolved and suspended solid PAHs in water; 15 PAHs (listed in Table 2).

c Air samples were collected in September

d NA (due to sampling failure)

NS and MW represent urban sites, and KS and KN represents suburban sites as illustrated in Figure 1.

TSP and Foc are concentration of total suspended particle in air and fraction of organic carbon, respectively.

**Table 2.** Concentration range (median) of target PAH compounds measured for each medium at the two sites (urban and suburban).

PAHs		Air (ng/m^3^)		Soil (ng/g dw)	Sediment (ng/g dw)	Water (ng/L)		Plant leave (ng/g dw.)	
		Gaseous	particulate			dissolved	SS	deciduous	coniferous

*N (sample number)* =		9	9	6	10	10	10	8	8
Naphthalaene	NA	NQ	0.031-0.20 (0.092)	20-44 (28)	5.6-28 (14)	NQ	NQ	11-62 (14)	14-450 (160)
2-methylnaphthalene	2-MeNA	NQ	0.016-0.11 (0.059)	10-26 (18)	3.3-19 (9.3)	NQ	NQ	3.4-32 (6.5)	18-170 (71)
1-methylnaphthalene	1-MeNA	NQ	0.008-0.06 (0.028)	4.9-14 (8.6)	1.7-8.7 (4.3)	NQ	NQ	2.1-22 (3.1)	5.1-110 (23)
Biphenyl	BI	NQ	0.02-0.078 (0.034)	5.3-11 (8.9)	3.4-8.2 (5.4)	NQ	NQ	3.7-37 (9.5)	12-290 (24)
2,6-dimethylnaphthalene	DiNA	NQ	0.007-0.053 (0.024)	5.2-11 (8.7)	2.6-15 (7.8)	NQ	NQ	0.3-15 (3.3)	3.2-23 (15)
Acenaphthylene	AC	NQ	0.006-0.19 (0.035)	1.8-3.0 (2.5)	0.40-1.1 (0.7)	NQ	NQ	0.50-23 (2.2)	2.9-180 (7.3)
Acenaphthene	ACE	NQ	0.001-0.022 (0.0070)	1.6-2.5 (1.7)	0.60-3.5 (1.1)	NQ	NQ	0.40-7.0 (1.6)	0.50-37 (4.6)
2,3,5-trimethyl-naphthalene	TriNA	NQ	0.004-0.026 (0.013)	2.1-7.4 (4.5)	1.8-11 (3.6)	NQ	NQ	0.80-17 (2.4)	4.7-140 (17)
Fluorene	FL	NQ	0.005-0.24 (0.038)	2.8-8.0 (4.3)	1.8-8.2 (3.8)	NQ	NQ	2.4-81 (8.1)	28-120 (37)
Phenanthrene	PH	2.3-14 (6.8)	0.058-3.2 (0.48)	53-85 (69)	11-38 (19)	1.6-9.4 (4.2)	0.26-1.5 (0.90)	16-810 (62)	130-510 (210)
Anthracene	AN	0.053-1.0 (0.32)	0.006-0.16 (0.044)	1.7-4.4 (2.8)	1.2-5.8 (2.6)	0.14-5.5 (0.27)	0.024-0.09 (0.051)	0.70-20 (3.6)	0.20-10 (3.6)
1-methylphenanthrene	MePH	0.25-0.91 (0.55)	0.009-0.29 (0.089)	8.6-14 (9.5)	1.6-6.0 (3.3)	0.28-1.1 (0.49)	0.061-3.4 (0.18)	2.3-76 (9.2)	7.4-52 (20)
Fluoranthene	FLR	1.2-3.6 (2.0)	0.078-4.2 (0.99)	99-150 (110)	21-54 (32)	0.35-2.3 (0.91)	0.11-1.0 (0.57)	33-410 (110)	24-200 (79)
Pyrene	PY	0.80-3.2 (1.7)	0.092-3.6 (0.93)	63-120 (79)	19-48 (28)	0.30-3.6 (1.1)	0.16-1.3 (0.42)	17-210 (57)	20-170 (62)
Benz[a]anthracene	BaA	0.004-0.088 (0.029)	0.050-1.67 (0.61)	20-57 (33)	8.0-23 (13)	0.035-3.4 (0.13)	0.095-0.37 (0.16)	1.6-17 (6.0)	0.80-12 (6.1)
Chrysene	CH	0.027-0.33 (0.10)	0.12-3.2 (1.1)	64-140 (78)	12-26 (18)	0.090-0.87 (0.16)	0.051-0.60 (0.31)	16-97 (24)	5.0-59 (34)
Benzo[b]fluoranthene	BbF	0.002-0.14 (0.010)	0.25-5.2 (2.2)	88-230 (130)	19-42 (30)	0.046-1.9 (0.16)	0.058-0.70 (0.28)	5.2-44 (11)	1.3-19 (11)
Benzo[k]fluoranthene	BkF	nd-0.035 (0.0040)	0.057-1.4 (0.52)	18-65 (30)	5.0-12 (7.7)	0.012-1.2 (0.097)	0.012-0.20 (0.082)	1.2-11 (2.3)	0.30-4.5 (2.6)
Benzo[e]pyrene	BeP	nd-0.057 (0.005)	0.13-2.0 (1.0)	38-100 (55)	9.4-21 (14)	0.015-0.58 (0.082)	0.040-0.40 (0.15)	3.2-19 (6.1)	0.70-8.3 (4.9)
Benzo(a)pyrene	BaP	nd-0.061 (0.0050)	0.067-2.3 (1.1)	22-75 (39)	7.5-22 (15)	0.037-0.48 (0.30)	0.031-0.22 (0.081)	1.0-11 (2.6)	0.30-3.5 (1.9)
Perylene	PE	Nd-0.017 (0.0010)	0.011-1.7 (0.14)	4.5-14 (8.0)	8.0-28 (12)	0.036-0.30 (0.074)	0.042-0.61 (0.079)	nd-1.3 (0.50)	0.30-0.80 (0.40)
Indeno[1,2,3-cd]pyrne	IP	Nd-0.082 (0.0030)	0.19-7.5 (1.3)	90-290 (150)	24-52 (35)	0.040-0.87 (0.28)	0.012-0.52 (0.21)	1.5-20 (6.4)	0.80-7.0 (4.8)
Benzo[ghi]perylene	BPE	ND-0.071 (0.0060)	0.22-5.2 (1.3)	64-240 (96)	18-50 (25)	0.036-0.88 (0.34)	0.082-0.70 (0.31)	1.9-17 (12)	1.1-12 (4.9)
Dibenzo[a,h]anthracene	DA	ND-0.012 (0.0050)	0.033-0.91 (0.19)	23-64 (37)	7.4-15 (10)	0.11-0.37 (0.27)	0.024-0.13 (0.063)	0.50-2.9 (0.80)	nd-1.2 (0.70)

NQ: not quantified
